# Molecular phylogeny of the forensically important genus *Cochliomyia* (Diptera: Calliphoridae)

**DOI:** 10.3897/zookeys.609.8638

**Published:** 2016-08-08

**Authors:** Sohath Yusseff-Vanegas, Ingi Agnarsson

**Affiliations:** 1 Department of Biology, University of Vermont, 109 Carrigan Drive, Burlington, VT 05405, USA

**Keywords:** Forensic entomology, Caribbean region, habitat preferences, Cochliomyia minima, Cochlioyia aldrichi, Cochliomyia macellaria, Cochliomyia hominivorax

## Abstract

*Cochliomyia* Townsend includes several abundant and one of the most broadly distributed, blow flies in the Americas, and is of significant economic and forensic importance. For decades, *Cochliomyia hominivorax* (Coquerel) and *Cochliomyia macellaria* (Fabricius) have received attention as livestock parasites and primary indicator species in forensic entomology. However, *Cochliomyia minima* Shannon and *Cochliomyia aldrichi* Del Ponte have only been subject to basic taxonomy and faunistic studies. Here we present the first complete phylogeny of *Cochliomyia* including numerous specimens per species, collected from 13 localities in the Caribbean. Four genes, the mitochondrial COI and the nuclear EF-1α, 28S rRNA, and ITS2, were analyzed. While we found some differences among gene trees, a concatenated gene matrix recovered a robustly supported monophyletic *Cochliomyia* with *Compsomyiops* Townsend as its sister group and recovered the monophyly of *Cochliomyia hominivorax*, *Cochliomyia macellaria* and *Cochliomyia minima*. Our results support a close relationship between *Cochliomyia minima* and *Cochliomyia aldrichi*. However, we found *Cochliomyia aldrichi* containing *Cochliomyia minima*, indicating recent speciation, or issues with the taxonomy of the group. We provide basic information on habitat preference, distribution and feeding habits of *Cochliomyia minima* and *Cochliomyia aldrichi* that will be useful for future forensic studies in the Caribbean.

## Introduction

*Cochliomyia* Townsend is endemic to the Americas and includes only four species: *Cochliomyia minima* Shannon, *Cochliomyia aldrichi* Del Ponte, *Cochliomyia macellaria* (Fabricius) and *Cochliomyia hominivorax* (Coquerel). All of them are flesh eaters during their larval stage and are locally abundant. In particular, *Cochliomyia macellaria* is one of the most broadly distributed blow flies in the New World (Whitworth 2010). These species vary in habitat preference, feeding habits, dispersal abilities, and morphology among the species (Hall 1948, Whitworth 2010). For instance, *Cochliomyia aldrichi*, *Cochliomyia minima* and *Cochliomyia macellaria* are primarily carrion feeders, while, *Cochliomyia hominivorax* is an obligate parasite of mammals (Hall 1948, Stevens and Wallman 2006, McDonagh et al. 2009, McDonagh and Stevens 2011).

*Cochliomyia hominivorax* and *Cochliomyia macellaria* have been intensely studied due to their commercial and forensic importance. *Cochliomyia macellaria* is one of the most forensically important species commonly found on decomposing remains. This species is considered important for post mortem interval estimations (Smith 1986, Byrd and Castner 2010) being among the first species to colonize corpses. In contrast, *Cochliomyia hominivorax* is an obligate parasite with its larvae producing myiasis and feeding on living tissue (Hall 1948, Guimaraes et al. 1983). This species is one of the most important insect pests of livestock in the Neotropics causing economic losses of billions of dollars every year (Vargas-Terán et al. 2005). Both species are common throughout the year in tropical, warm and humid areas (Hall 1948). *Cochliomyia macellaria* can be found in temperate climates from Canada to Argentina during the summer months (Whitworth 2010). *Cochliomyia hominivorax* initially ranged from southern United States to northern Argentina (Guimaraes et al. 1983) but has been eradicated from North America, Central America, Puerto Rico and the Virgin Islands (Vargas-Terán et al. 2005). It is worth noting that in 1988 this species was introduced in Libya and it was successfully eradicated in 1992 based on the sterile insect technique (SIT). This was the major international effort and avoid a major disaster for the livestock industry of Africa and Southern Europe (Lindquist et al. 1992). Despite those successfully eradications *Cochliomyia hominivorax* continues to be an economically important pest in South America and parts of the Caribbean (Vargas-Terán et al. 2005).

The other two congeners, *Cochliomyia minima* and *Cochliomyia aldrichi*, are poorly known and research has been limited to descriptive morphology and faunistics (Hall 1948, Dear 1985, Whitworth 2010). These two species are restricted to the West Indies and *Cochliomyia aldrichi* has been reported in the Florida Keys (Whitworth 2010). Dear (1985) listed *Cochliomyia minima* for the Florida Keys, however Whitworth (2010) concluded that Dear mistakenly identified one *Cochliomyia aldrichi* specimen as *Cochliomyia minima*. Forensically important insects in the Caribbean are generally understudied and these two species have not played an important role in forensic entomology. Yet, due to their abundance and broad distribution in this region, including Cuba, Dominican Republic, Jamaica, Puerto Rico, Virgin Islands, Bahamas and Cayman Islands (Hall 1948, Dear 1985, Whitworth 2010) they have an enormous forensic potential. For example, recent studies conducted in Puerto Rico showed that *Cochliomyia minima* is abundant and widely distributed on the island, and that adults are attracted to, and feed on, carrion (Yusseff-Vanegas 2014).

Although the adult morphology of the four species is well known (Hall 1948, Dear 1985, Whitworth 2010), studies on the relationship among *Cochliomyia* species have not been conducted yet. Morphological studies have provided synapomorphies of *Cochliomyia* that clearly diagnose it from all other Calliphoridae (Hall 1948; Dear 1985; Whitworth 2010). These include short and filiform palpus and phallus with extremely elongated paraphallus and a complex distiphallus (Dear 1985, Figs 37–44). Prior studies on the relationships among Calliphoridae (Stevens 2003, Harvey et al. 2008, McDonagh and Stevens 2011) and the subfamily Chrysomyinae (Singh and Wells 2011), including *Cochliomyia macellaria* and *Cochliomyia hominivorax*, supported *Cochliomyia* monophyly, and placed it as sister to *Compsomyiops* Townsend. However, the monophyly of the genus has not been formally tested with thorough sampling of all species, and the relationships among its species remain unknown. Furthermore, DNA-based methods can provide reliable identification of specimens by non-experts and will be particularly important for the identification of larval stages of *Cochliomyia minina* and *Cochliomyia aldrichi* that remain poorly known. For example, only the third instar of *Cochliomyia minima* has been described (Yusseff-Vanegas 2014).

Here we provide a robust phylogenetic hypothesis of *Cochliomyia* based on four genes sequenced from 38 individuals collected throughout the Caribbean, including for the first time molecular data about *Cochliomyia minima* and *Cochliomyia aldrichi*. Our main goals are to test the monophyly of this genus and the validity of, and relationships among, its species.

## Methods

### Specimens and DNA extraction

A total of 44 specimens were included in this study, 38 representing the ingroup plus six outgroup species [*Chrysomya megacephala* (Fabricius), *Chrysomya rufifacies* (Macquart), *Hemilucilia* sp., *Lucilia cuprina* (Wiedemann), *Compsomyiops fulvicrura* (Robineau-Desvoidy) and *Compsomyiops callipes* (Bigot)]. All sequences used here are new except for *Compsomyiops fulviclura* and *Compsomyiops callipes* (Table 1). The specimens were collected in the Caribbean (Jamaica, Cuba, Dominican Republic, Puerto Rico, Saint Barts Martinique and Dominica) from 2011 to 2013 and in the following countries, Colombia (2014), Florida (2013) and Mexico (2010 and 2012) (Table 1). All specimens were killed and preserved in 95% ethanol and stored at -20 °C. The adults were examined with a Leica MZ16 stereomicroscope and identified using the Whitworth (2010) keys. The DNA was isolated from thoracic muscle or two legs of each individual with the QIAGEN DNeasy Tissue Kit (Qiagen, Inc., Valencia, CA). Voucher specimens were deposited at the UVM Natural History Museum (in the Zadock Thompson Zoological Collections) and sequences were submitted to GenBank.

**Table 1.** Specimen details, collection information and GenBank accession numbers.

Species name – Voucher Number	Location	*CO1*	*EF-1*α	*ITS2*	*28S rRNA*
*Cochliomyia macellaria CO002*	Colombia, El refugio Dry Forest	KX529522	KX529616	KX529574	KX529487
*Cochliomyia macellaria CO010*	Colombia, Choco, Jardín botánico del Pacífico	KX529545	KX529617	KX529575	KX529488
*Cochliomyia macellaria CO017*	Colombia, Santander, Chipatá, Finca el Castillo	KX529543	KX529618	KX529576	KX529489
Cochliomyia macellaria ME015*	Mexico, Torreon, Coahuila	KX529546	KX529629	KX529588	KX529492
*Cochliomyia macellaria FL006*	USA, Florida, Everglades National Park, Northeast	KX529535	KX529623	KX529581	KX529503
*Cochliomyia macellaria JA002*	Jamaica, Marshall’s Pen House	KX529538	KX529624	KX529582	KX529502
*Cochliomyia macellaria CU018*	Cuba, Pinar del Rio, Viñales Nacional Park	KX529526	KX529620	KX529578	KX529499
*Cochliomyia macellaria CU014*	Cuba, Pinar del Rio, Viñales Nacional Park	KX529541	KX529619	KX529577	KX529497
*Cochliomyia macellaria DR134*	Dominican Republic, Puerto Plata	KX529527	KX529622	KX529580	KX529504
*Cochliomyia macellaria DR010*	Dominican Republic, El Morro, Monte Cristi	KX529536	KX529621	KX529579	KX529496
*Cochliomyia macellaria PR129*	Puerto Rico, Vieques, Monte Pirata	KX529542	-	KX529591	KX529501
*Cochliomyia macellaria PR128*	Puerto Rico, Vieques, Monte Pirata	KX529540	-	KX529590	KX529494
*Cochliomyia macellaria PR121*	Puerto Rico, Trujillo Alto, Ciudad Universitaria	KX529544	KX529630	KX529589	KX529500
*Cochliomyia macellaria M112*	Puerto Rico, Isla de Mona, Los Caobos	KX529528	-	KX529587	KX529493
*Cochliomyia macellaria M081*	Puerto Rico, Isla de Mona, Los Caobos	KX529537	KX529628	KX529586	KX529498
*Cochliomyia macellaria M077*	Puerto Rico, Isla de Mona, Bajuras - Cerezos	KX529539	KX529627	KX529585	KX529495
*Cochliomyia macellaria LA142*	Saint Barts, Colombier Deciduos Dry Forest	KX529523	KX529631	KX529592	-
*Cochliomyia macellaria LA096*	Martinique, Cap de Macré Coastal Forest	KX529524	KX529626	KX529584	KX529491
*Cochliomyia macellaria LA071*	Dominica, Middleham Falls Trail	KX529525	KX529625	KX529583	KX529490
*Cochliomyia aldrichi M080*	Puerto Rico, Isla de Mona, Near Cueva Portugues	KX529529	KX529605	KX529563	KX529513
*Cochliomyia aldrichi M085*	Puerto Rico, Isla de Mona, Los Caobos	KX529530	KX529606	KX529564	KX529515
*Cochliomyia aldrichi M086*	Puerto Rico, Isla de Mona, Camino del Indio	KX529531	KX529607	KX529565	KX529514
*Cochliomyia aldrichi M103*	Puerto Rico, Isla de Mona, Los Caobos	KX529532	KX529608	KX529566	KX529516
*Cochliomyia aldrichi M105*	Puerto Rico, Isla de Mona, Near Cueva Portugues	KX529533	KX529609	KX529567	KX529518
*Cochliomyia aldrichi M107*	Puerto Rico, Isla de Mona, Near Cueva Portugues	KX529534	KX529610	KX529568	KX529517
*Cochliomyia minima CU046*	Cuba, Guantanamo, Alejandro de Humboldt National Park	KX529547	KX529633	KX529595	KX529510
*Cochliomyia minima CU022*	Cuba, Pinar del Rio, Viñales National Park	KX529549	KX529632	KX529593	KX529511
*Cochliomyia minima CU023*	Cuba, Pinar del Rio, Viñales National Park	KX529550	-	KX529594	KX529508
*Cochliomyia minima DR136*	Dominican Republic, Puerto Plata	KX529548	KX529635	KX529597	KX529509
*Cochliomyia minima DR055*	Dominican Republic, Haitises National Park	KX529552	KX529634	KX529596	KX529507
*Cochliomyia minima PR141*	Puerto Rico, Loiza, Mangrove area	KX529551	-	KX529600	KX529512
*Cochliomyia minima PR132*	Puerto Rico, Loiza, Mangrove area	KX529553	KX529636	KX529598	-
*Cochliomyia minima PR133*	Puerto Rico, Vieques, Monte Pirata	KX529554	KX529637	KX529599	KX529506
*Cochliomyia hominivorax CO001*	Colombia, El refugio Dry Forest	-	KX529611	KX529569	KX529482
*Cochliomyia hominivorax CU020*	Cuba, Pinar del Rio, Viñales Nacional Park	-	KX529612	KX529570	KX529483
*Cochliomyia hominivorax CU033*	Cuba, Pinar del Rio, Viñales Nacional Park	KX529556	KX529613	KX529571	KX529484
*Cochliomyia hominivorax DR042*	Dominican Republic, Rabo de Gato	KX529557	KX529614	KX529572	KX529485
*Cochliomyia hominivorax DR105*	Dominican Republic, East National Park, Yuma	KX529558	KX529615	KX529573	KX529486
*Chrysomya megacephala FL003*	USA, Florida, Everglades National Park, Northeast	KX529521	KX529603	KX529561	KX529480
*Chrysomya rufifacies CU004*	Cuba, Granma: Turquino National Park	KX529555	KX529604	KX529562	KX529481
*Hemilucilia sp. CO018*	Colombia, Santander, Chipatá, Finca el Castillo	KX529560	KX529638	KX529601	KX529519
*Lucilia cuprina PR073*	Puerto Rico, Trujillo Alto, Ciudad Universitaria	KX529559	KX529639	KX529602	KX529520
*Compsomyiops fulvicrura*	As Published (Kutty et al. 2008)	FJ025607	FJ025667	-	FJ025504
*Compsomyiops callipes*	As Published (Wells and Sperling 2001)	AF295549	-	-	-

*The sample from Mexico was collected by Fabián García Espinoza from *Universidad Antonio Narro Unidad Laguna*.

### PCR amplification and sequencing

We amplified regions of three nuclear loci: the protein coding elongation factor-1 alpha (EF-1α), the ribosomal 28S, and internal transcribed spacer 2 (ITS2), plus the mitochondrial protein coding cytochrome oxidase I (COI). The primer sequences are listed in Table 2. Protocols for COI reactions included an initial denaturation step of 95 °C for 2 min, followed by 35 cycles of 95 °C for 30 s, 44 °C for 45 s and 72 °C for 45 s, and a final elongation step of 72 °C for 10 min (Agnarsson et al. 2007). For ITS2 an initial denaturation step of 94 °C for 2 min was followed by 38 cycles of 94 °C for 30 s, 44 °C for 35 s and 72 °C for 30 s, and a final elongation step of 72 °C for 3 min (Agnarsson 2010). For EF-1α an initial denaturation of 95 °C for 5 min was followed by 35 cycles of 94 °C for 30 s, 55 °C for 35 s and 72 °C for 1 min, and a final elongation step of 72 °C for 10 min (McDonagh et al. 2009). For 28S rRNA initial denaturation of 94 °C for 5 min was followed by 35 cycles of 93 °C for 1 min, 60 °C for 1 min and 72 °C for 2 min, and a final elongation step of 72 °C for 3 min (Friedrich and Tautz 1997). Amplified fragments were sequenced in both directions by University of Arizona Genetics Core. Sequences were interpreted from chromatograms using Phred (Green and Ewing 2002) and Phrap (Green 1999, Green and Ewing 2002) using the Chromaseq module (Maddison and Maddison 2010a) in the evolutionary analysis program Mesquite 3.03 (Maddison and Maddison 2010b) with default parameters. The sequences were then proofread by examining chromatograms by eye. Alignments were done using MAFFT (Katoh et al. 2002) through the online portal EMBL-EBI. The gene matrices were then concatenated in Mesquite 3.03 (Maddison and Maddison 2010b) and the full aligned data set is 3368 bp.

**Table 2.** PCR primers use in this study.

Gene	Primer name	Sequence (5’ to 3’)	Source
COI	LCO1490	GGTCAACAAATCATAAAGATATTGG	Folmer et al. (1994)
	CI-N-2776	GGATAATCAGAATATCGTCGAGG	Hedin and Maddison (2001)
EF-1α	B1	CCCATYTCCGGHTGGCACGG	McDonagh et al. (2009)
	C1	CTCTCATGTCACGDACRGCG	McDonagh et al. (2009)
28S	D1.F	CCCCCTGAATTTAAGCATAT	Friedrich and Tautz (1997)
	D35.486.R	TCGGAAGGAACCAGCTACTA	Friedrich and Tautz (1997)
ITS	ITS4	TCCTCCGCTTATTGATATGC	White et al. (1990)
	ITS5.8	GGGACGATGAAGAACGCAGC	Agnarsson (2010)

### Phylogenetic analysis

We partitioned each gene and codon position for a total of eight partitions that were exported from Mesquite for model choice and the appropriate models were chosen using jModeltest v2.1.4 (Posada and Crandall 1998), and the AIC criterion (Posada and Buckley 2004). The corresponding model of evolution was used for the Bayesian analysis: GTR + Γ + I for 28S, ITS2 and COI3rd, GTR + Γ for COI1st, COI2nd, EF-1α3rd, HKY + Γ for EF-1α2nd and F81 for EF-1α1st. We ran the MC^3^ (Metropolis Coupled Markov Chain Monte Carlo) chain in MrBayes v3.2.3 (Huelsenbeck and Ronquist 2001) through the online portal Cipres Science Gateway v3.3 (Miller et al. 2010). The analysis was run for 30.000.000 generations, sampling every 1000 generations. Chain stationary, ESS, and appropriate burnin was verified using Tracer 1.6 (Rambaut and Drummond 2009). Maximum likelihood (ML) analysis of the concatenated matrix was done in Garli (Zwickl 2006) using the same partitioning scheme and models.

## Results

**Figure 1.**
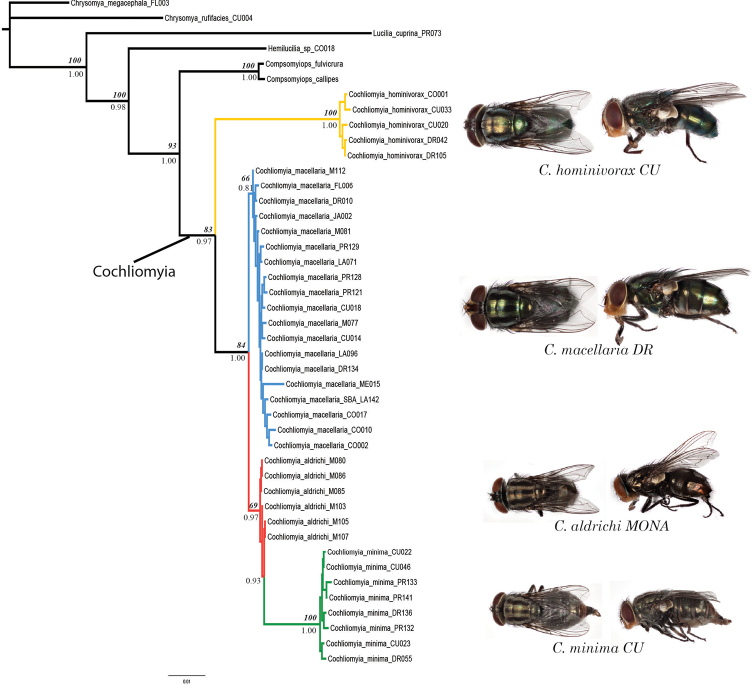
Phylogenetic relationship within *Cochliomyia* (ingroup) based on partitioned Bayesian analysis of the combined gene (COI, EF-1α, 28S rRNA and ITS2) data set. Branch support values: normal fond, Bayesian posterior probability; bold-italic font, maximum likelihood percentage bootstrap. Each color represents different species.

The phylogenetic analyses of the concatenated matrix, either using Bayesian or maximum likelihood approaches, recovered a generally well supported monophyletic *Cochliomyia* (Fig. 1). *Cochliomyia macellaria*, *Cochliomyia hominivorax* and *Cochliomyia minima* were recovered as monophyletic, while *Cochliomyia aldrichi* was recovered as paraphyletic.

Independent analyses of 28S and ITS2 supported the monophyly of *Cochliomyia*, while COI and EF-1α recovered it as a paraphyletic group (Suppl. material 1). At the species level, EF-1α and 28S had limited signal and did not distinguish between *Cochliomyia minima* and *Cochliomyia aldrichi*. COI recovered the monophyly of *Cochliomyia minima*, but did not resolve relationships among *Cochliomyia aldrichi* and *Cochliomyia macellaria*. ITS2 fully resolved the relationships within *Cochliomyia*, and is the only gene that recovered the monophyly of *Cochliomyia aldrichi*. Despite of the incongruence detected among the four gene trees, they all recovered monophyletic *Cochliomyia hominivorax* and three of the four genes (COI, 28S and ITS2) strongly supported a monophyletic *Cochliomyia hominivorax* as sister to the other three species.

The concatenated dataset yielded a topology supporting a close relationship between *Cochliomyia minima* and *Cochliomyia aldrichi* which is congruent with the current taxonomy and indicates *Cochliomyia macellaria* as the sister lineage of these two.

## Discussion

We present the first species complete phylogeny of the genus *Cochliomyia* including samples collected throughout the Caribbean from 13 different localities (Table 1). The concatenated matrix recovered a monophyletic *Cochliomyia*, partially resolved relationships among its species and recovered *Compsomyiops* as its sister group (Fig. 1), in congruence with prior studies (McDonagh and Stevens 2011, Singh and Wells 2011).

Independent gene trees did not yield fully congruent relationships among species, unsurprising as genes have independent histories. Two nuclear genes, 28S and ITS2 (adjacent loci), strongly supported the monophyly of *Cochliomyia* while the other two genes, COI and EF-1α did not. These results differ from McDonagh (2009), where EF-1α and COI strongly supported the monophyly of *Cochliomyia*, while 28S recovered *Cochliomyia* as paraphyletic. However, McDonagh (2009) included only two of the species of *Cochliomyia* represented by one specimen each. The differences between the studies could be due to a variety of taxon sampling issues, where our sampling was designed specifically to test monophyly and relationships among *Cochliomyia* species.

The monophyly of *Cochliomyia hominivorax* is supported in all analyses, however, independent gene trees were not congruent with regards to other species. The relatively slowly evolving nuclear genes EF-1α and 28S supported *Cochliomyia macellaria* but failed to distinguish between *Cochliomyia minima* and *Cochliomyia aldrichi*. The relatively rapidly evolving COI “DNA barcode” was found suitable for species identification and delineation (Hebert et al. 2003). COI was the only gene that recovered the monophyly of *Cochliomyia minima*, however, COI did not resolve relationships among specimens of *Cochliomyia aldrichi* and *Cochliomyia macellaria*. This is surprising as these species are clearly identifiable based on morphological characteristics (Hall 1948, Whitworth 2010). Other studies also reported similar results where COI failed to distinguish among some closely related calliphorids (Wallman and Donnellan 2001, Nelson et al. 2007, Whitworth et al. 2007, Harvey et al. 2008, DeBry et al. 2013, Whitworth 2014), a result that has been attributed to incomplete lineage sorting. Results from COI, EF-1α, and 28S combined suggested *Cochliomyia aldrichi* as sister to *Cochliomyia macellaria*, instead of to *Cochliomyia minima* as we would expect based on morphological characteristics. Based on these results we opted to add the rapidly evolving nuclear marker, ITS2 to help resolve species level relationships (Nelson et al. 2007, Agnarsson 2010). ITS2 was the only gene that recovered *Cochliomyia aldrichi* as a monophyletic group and supported *Cochliomyia minima* as its sister lineage.

Despite the incongruence detected between the four genes, a concatenate matrix recovered the monophyly of *Cochliomyia hominivorax*, *Cochliomyia macellaria* and *Cochliomyia minima*, and supported the monophyly of *Cochliomyia minima* plus *Cochliomyia aldrichi*, mostly congruent with the current taxonomy. However, we found that *Cochliomyia minima* is nested within *Cochliomyia aldrichi*. That one species is paraphyletic with respect to another is not unexpected and does not necessarily refute their species status. The non-monophyly of *Cochliomyia aldrichi* is surprising in that all specimens included in this study were collected from the tiny Mona Island (22 square miles). This indicates incomplete lineage sorting, or possibly recent speciation, rather than other processes like gene flow among species (given Mona’s isolation, expectation of panmixia among *Cochliomyia aldrichi* on the tiny island, and absence of *Cochliomyia aldrichi* from other islands sampled). In contrast, *Cochliomyia macellaria* and *Cochliomyia minima* are present on most of the islands (Table 1) and the populations in different islands do not show any geographic structure (Fig. 1), indicating a constant gene flow among populations through migration.

**Figure 2.**
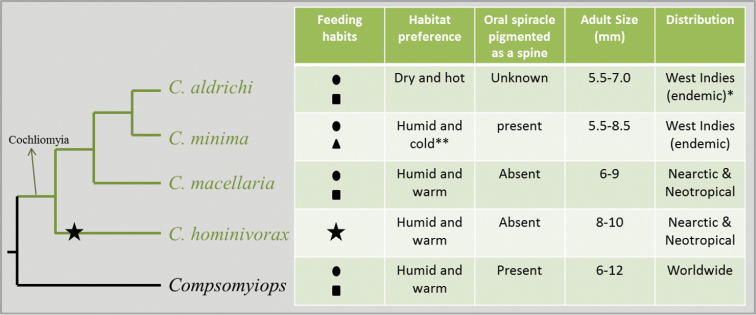
Variability in feeding habits, habitat preference and morphology within *Cochliomyia*. *Cochliomyia aldrichi has been reported in the Florida Keys Islands. **We refer to temperatures around 10–15 °C. ● Carrion feeder; ▴ primary facultative parasite; ■ secondary facultative parasite; ★ obligate parasite.

The variability in feeding habits, habitat preference and morphology within *Cochliomyia* is considerable (Fig. 2). In feeding habits, *Cochliomyia aldrichi*, *Cochliomyia minima* and *Cochliomyia macellaria* share similar behaviors. They are primarily carrion feeders, commonly found on decomposing cadavers. However, they are also capable of producing myiasis in open wounds as secondary facultative parasites under certain conditions or as primary facultative parasites as in the case of *Cochliomyia minima*, (Hall 1948, Dear 1985). In contrast, *Cochliomyia hominivorax* is an obligate parasite of mammals never found in decaying meats (Hall 1948, Stevens and Wallman 2006, McDonagh et al. 2009, McDonagh and Stevens 2011, but see Brody and Knipling 1943). Several authors have studied the evolution of parasitism within Calliphoridae and have concluded that the parasitic behavior in this family evolved independently several times (Stevens and Wallman 2006, McDonagh and Stevens 2011, Singh and Wells 2011). Within *Cochliomyia*, we conclude that parasitism evolved once in *Cochliomyia hominivorax*, since the congeners are carrion feeders, as are members of the sister group, *Compsomyiops* (Fig. 2).

The habitat preferences of *Cochliomyia hominivorax* and *Cochliomyia macellaria* are largely known (Hall 1948, Greenberg 1971, Smith 1986, Wells and Greenberg 1992, Byrd and Butler 1996, Byrd and Castner 2010, Koller et al. 2011), however, little is known about *Cochliomyia minima* and *Cochliomyia aldrichi*. In recent studies of *Cochliomyia minima* in Puerto Rico, Yusseff-Vanegas (2014) reported that *Cochliomyia minima* prefer highly humid areas and can tolerate relatively cool conditions at altitudes >800m, while this species is absent from extremely dry and hot areas. Similar results were found in Dominican Republic and Cuba where *Cochliomyia minima* was found abundantly in tropical and subtropical rain/moist forest even at altitudes >1300m, but absent from dry forest (unpublished data). These results supported the assumption that *Cochliomyia minima* prefer humid cool areas, however, more studies are needed to understand its habitat preferences. In contrast, *Cochliomyia aldrichi* seems to prefer hot dry areas, different from what we expected given the apparent recent divergence between *Cochliomyia minima* and *Cochliomyia aldrichi*. This is the case of recently divergent species like *Lucilia sericata* (Meigen) and *Lucilia cuprina* Wiedemann, and *Lucilia coeruleiviridis* Macquart and *Lucilia mexicana* Macquart that have similar habitat preferences (Stevens and Wall 1996, 1997, Whitworth 2006, Byrd and Castner 2010, Whitworth 2010, 2014). However, *Cochliomyia aldrichi* was found only on Mona Island, a subtropical dry forest with an average annual temperature of 27 °C (National Oceanic and Atmospheric Administration - NOAA) and low humidity through the year, strikingly different from *Cochliomyia minima*. Yet, similar results have been reported before for closely related species like *Chrysomya megacephala* and *Chrysomya pacifica* (Singh et al. 2011) which are characterized by very different habitat preferences (Kurahashi 1981, 1991). Despite we have extensively collected in Florida (Everglades and the Keys), Cuba, Puerto Rico and the Bahamas, where *Cochliomyia aldrichi* was previously reported (Whitworth 2010), we did not find this species. This could be explained by sampling bias as we only collected during the summer when precipitation and relative humidity are very high in the Caribbean. It is possible, for example, that *Cochliomyia aldrichi* may be seasonal, being present during the winter when conditions are generally drier and cooler in the Caribbean. Alternatively, our sampling might indicate the recent extinction of this species from areas outside Mona, nevertheless, further studies are necessary to test these alternative hypotheses.

Two of the four species, *Cochliomyia minima* and *Cochliomyia aldrichi* are Caribbean endemics while the other two are widespread (Figs 1–2). It is difficult to assess the biogeographical history of widespread species, however, we can conclude from our data that divergence between *Cochliomyia minima* and *Cochliomyia aldrichi* probably occurred in the Caribbean after the area was colonized. Island colonization is sometimes accompanied by a reduction in dispersal abilities and such processes may have led to reduced gene flow among islands, and promoted the formation of the Caribbean endemics. Further phylogeographic/phylogenomic studies including more taxa from the Caribbean and the continents are necessary to assess the colonization history of the genus and the possible secondary loss of dispersal ability in this group.

## Conclusions

We provide the first complete phylogeny of *Cochliomyia*, supporting its monophyly and placement within the subfamily Chrysomyinae. Given incongruence among gene trees and low level of information at the species level for slowly evolving genes, the resolution of the outstanding questions in *Cochliomyia* phylogeny will require more data rich approaches, such as those offered by NGS methods. Nevertheless, we advance knowledge on the phylogeny, distribution, and life history of these species that should prove useful in future research and in realizing the potential of these species as forensic insects.

## Supplementary materials

Supplementary material 1 Phylogenetic relationship within *Cochliomyia* (ingroup) based on a Bayesian analysis of nucleotide data from (a) 28S, (b) COI, (c) EF-1α and (d) ITS2 Data type: molecular data Explanation note: Numbers indicate posterior probability support values. This dataset is made available under the Open Database License (http://opendatacommons.org/licenses/odbl/1.0/). The Open Database License (ODbL) is a license agreement intended to allow users to freely share, modify, and use this Dataset while maintaining this same freedom for others, provided that the original source and author(s) are credited.Yusseff-Vanegas S, Agnarsson I
