# Supplementary figures and images for: Molecular phylogeny of the forensically important genus Cochliomyia (Diptera: Calliphoridae)
Source: Zookeys. 2016 Aug 8;(609):107–20. doi: 10.3897/zookeys.609.8638 (PMC4984421; doi:10.3897/zookeys.609.8638)

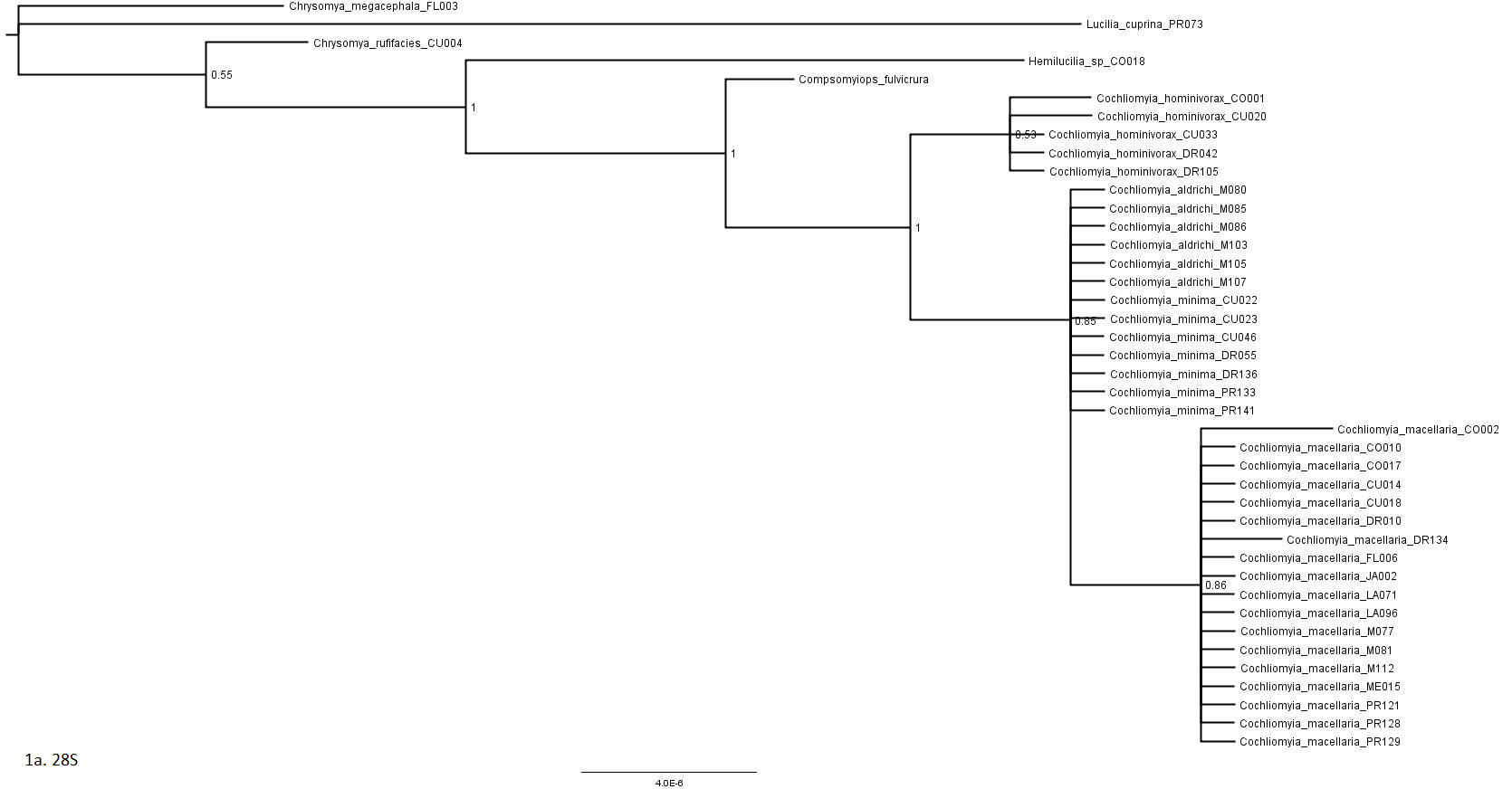

Supplement: Supplementary material 1 — Phylogenetic relationship within Cochliomyia (ingroup) based on a Bayesian analysis of nucleotide data from (a) 28S, (b) COI, (c) EF-1α and (d) ITS2 [file zookeys-609-107-s001.zip › 8638_RF_3_57818_A_95536.bmp]

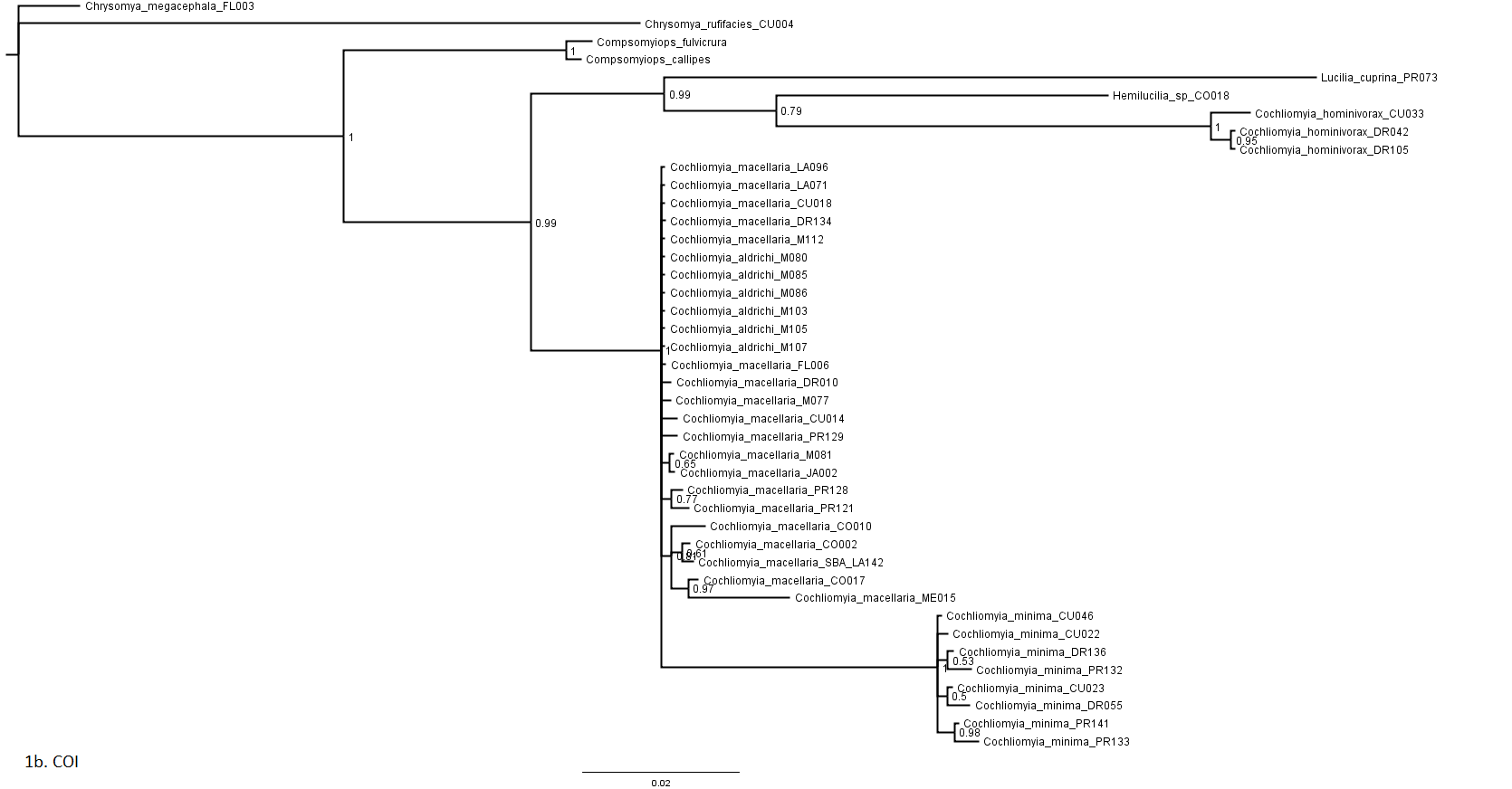

Supplement: Supplementary material 1 — Phylogenetic relationship within Cochliomyia (ingroup) based on a Bayesian analysis of nucleotide data from (a) 28S, (b) COI, (c) EF-1α and (d) ITS2 [file zookeys-609-107-s001.zip › 8638_RF_4_57818_A_95537.bmp]

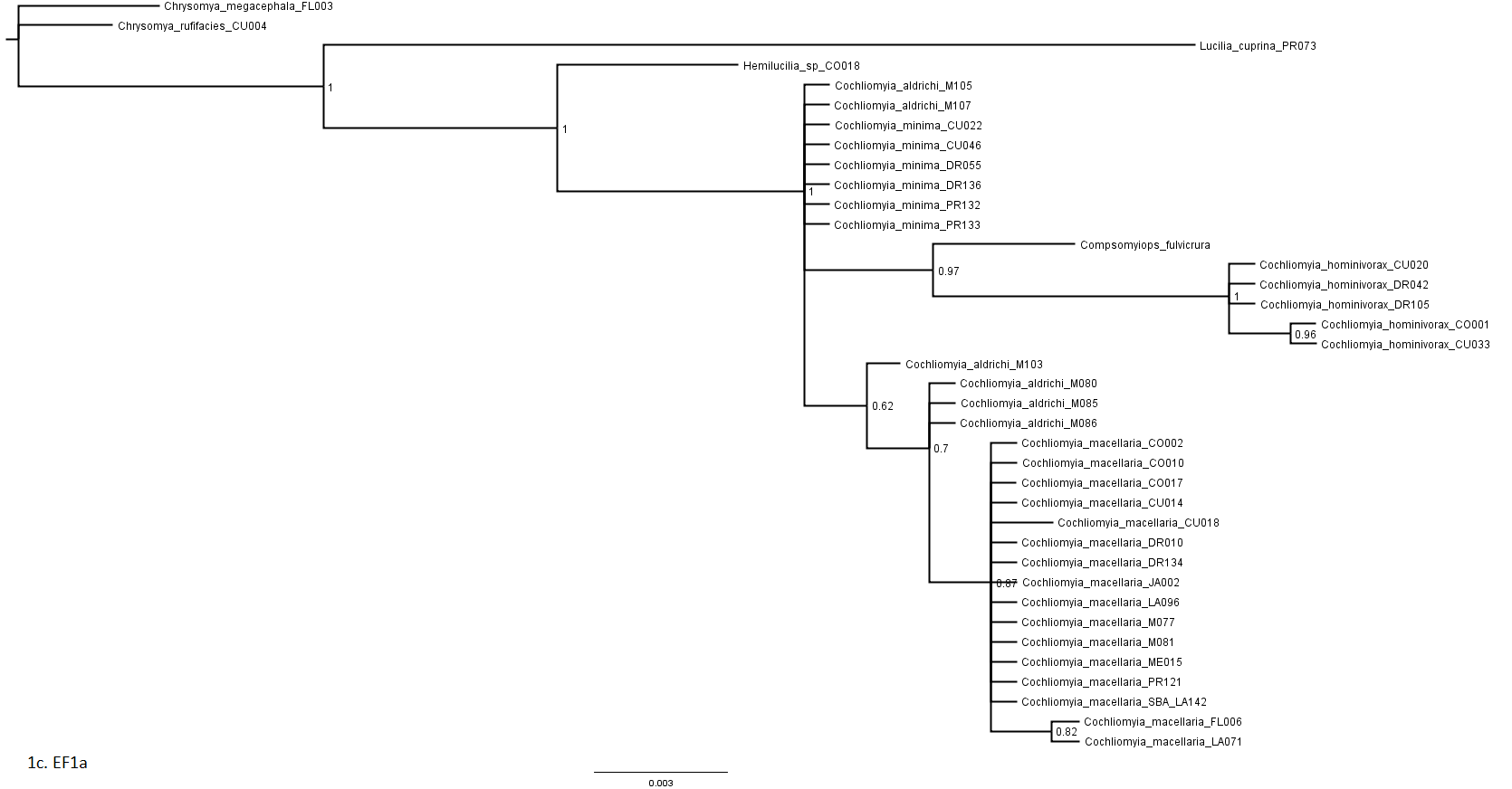

Supplement: Supplementary material 1 — Phylogenetic relationship within Cochliomyia (ingroup) based on a Bayesian analysis of nucleotide data from (a) 28S, (b) COI, (c) EF-1α and (d) ITS2 [file zookeys-609-107-s001.zip › 8638_RF_5_57818_A_95538.bmp]

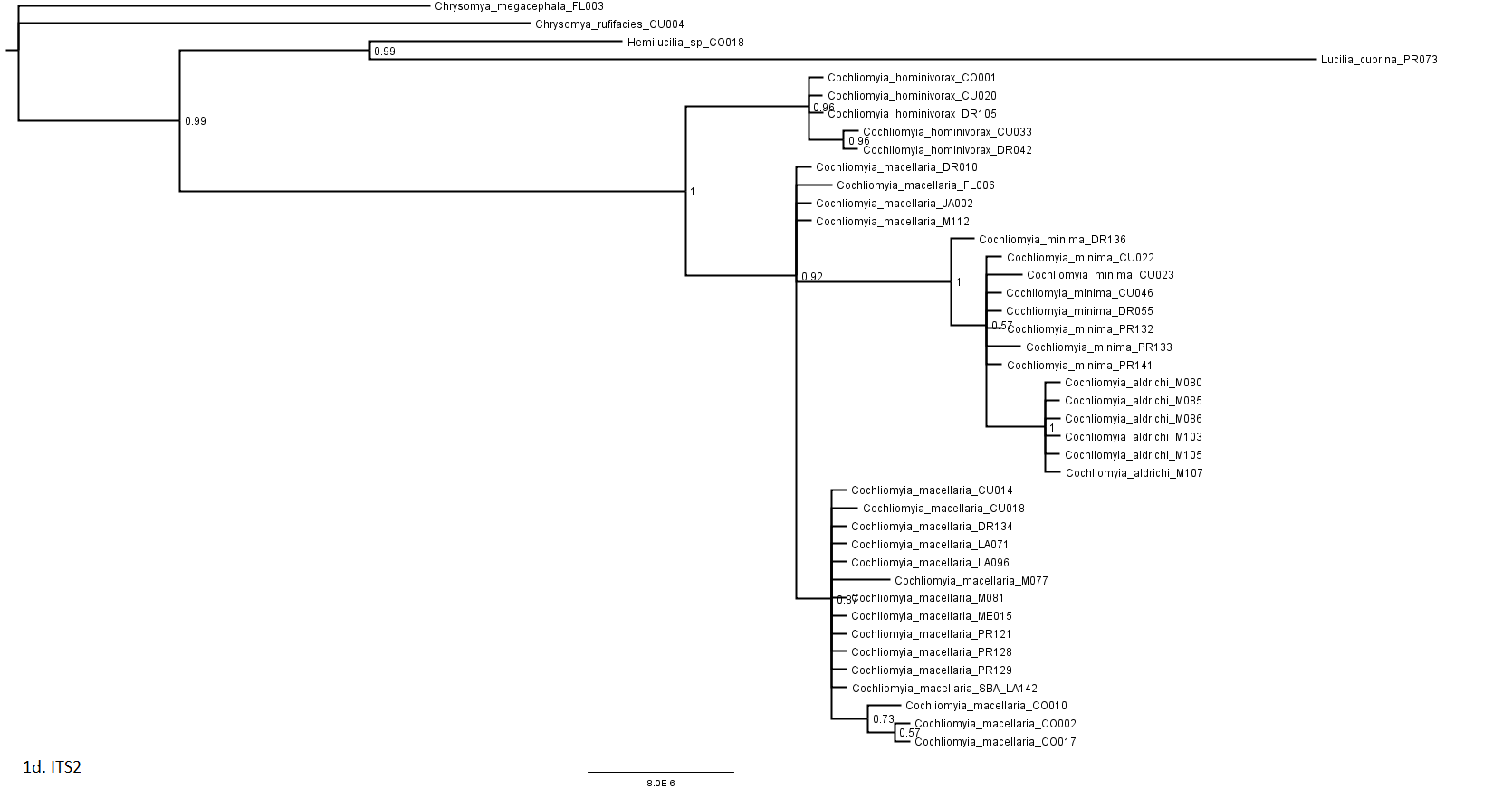

Supplement: Supplementary material 1 — Phylogenetic relationship within Cochliomyia (ingroup) based on a Bayesian analysis of nucleotide data from (a) 28S, (b) COI, (c) EF-1α and (d) ITS2 [file zookeys-609-107-s001.zip › 8638_RF_6_57818_A_95539.bmp]
